# Trends in Social Media Topics During COVID-19 Among Women Undergoing Assisted Reproductive Technology Treatments

**DOI:** 10.7759/cureus.11049

**Published:** 2020-10-19

**Authors:** Hanna R Perone, Hannah Stump, Alexandra Herweck, Hannah Levine, Adriana J Wong, Jose Carugno

**Affiliations:** 1 Department of Obstetrics, Gynecology, and Reproductive Sciences, University of Miami Miller School of Medicine, Miami, USA; 2 Department of Medical Education, Wright State University Boonshoft School of Medicine, Dayton, USA; 3 Department of Obstetrics and Gynecology, University of California Davis Health System, Sacramento, USA

**Keywords:** social media, instagram, covid-19, in vitro fertilization, qualitative analysis, infertility

## Abstract

Patients undergoing fertility treatments, such as in-vitro fertilization (IVF), face unique challenges both physically and mentally. With the emergence of the COVID-19 global pandemic, IVF patients began to face additional obstacles as hospitals and clinics shut down in compliance with recommendations for limiting exposure risk. In order to assess the impact of COVID-19 on IVF patients, we conducted a qualitative analysis using 563 public Instagram posts collected from three randomly selected days in March 2020. After the exclusion of 354 posts, thematic coding was used to analyze 209 posts. Five major themes were identified including (1) the medical and physical experience of IVF, (2) emotional spectrum, (3) sources of social support, (4) coping mechanisms, and (5) education on social media. Posts were categorized based on whether COVID-19 was discussed and theme frequencies were compared. The majority of patients impacted by the pandemic discussed setbacks to care, such as clinic closures. In addition, posts authored by those impacted by COVID-19 contained more negative emotions and fewer positive emotions compared to unaffected users. Despite an increase in setbacks and negative emotions, posts offering support nearly tripled in frequency highlighting the resilience of the IVF community. Our thematic analysis supports the need for careful consideration of the psychological and social effects of cycle cancellations on the IVF community. Experiences and sentiments revealed by this study should be considered when a successive pandemic or global emergency threatens IVF treatment protocols.

## Introduction

Approximately seven million couples seek medical care for infertility annually [1]. The process of undergoing medical treatment for infertility is challenging for women and requires significant cost, time, and tolerance for the physical effects of procedures and hormonal injections [2,3]. In-vitro fertilization (IVF) treatments often require multiple cycles to achieve pregnancy leading to emotional oscillations between hope and grief and thereby placing patients at increased risk of mental health conditions [4,5]. With the emergence of the COVID-19 pandemic, additional challenges arose as physicians, including infertility specialists, were confronted with achieving a balance between providing high-quality healthcare and minimizing patient risk. Broadly, the Centers for Disease Control (CDC) recommended that providers conduct telephone triage and utilize telemedicine technologies to minimize the risk of viral exposure [6]. Additionally, the American Society of Reproductive Medicine (ASRM) issued a national recommendation to postpone IVF cycles on March 17, 2020 [7]. Presently, the social and psychological impacts of these healthcare modifications on IVF patients are unknown. Therefore, the aim of this study was to utilize qualitative analysis of public Instagram posts to understand the experiences of patients undergoing, or who have previously undergone, IVF treatment during the COVID-19 global pandemic. 

## Materials and methods

Public Instagram posts using the hashtag, #ivfcommunity, were identified using a new Instagram account, ivf335, to avoid filtering of posts. Up to 200 posts from each day were included from three randomly selected dates in March following the ASRM’s recommendation (March 19, March 24, and March 25). All text, images, emojis, and relevant comments were extracted. Posts were excluded based on the following criteria: not in English (n=49), contained a video (n=23), unrelated to a patient’s IVF experience (n=96), reposted from another user (n=18), shared by a doctor/clinic or company (n=160), or included an advertisement (n=8). Thematic coding began with a four-member team who created the preliminary codebook using posts from one selected day [8]. Using open coding, five themes and sixteen corresponding subthemes were identified and agreed upon by all team members. After a low degree of discordance was noted during coding, the remaining posts were coded by two team members. For each post, coding groups identified themes applicable to each entry, the user’s presumed sex, and pregnancy status. Posts were classified as “COVID-19 posts” if COVID-19 and its implications were mentioned by the user. Though all posts analyzed were shared following the ASRM recommendation for cycle cancellation, the effect of COVID-19 was assessed by differences in theme content and frequencies of themes among COVID-19 versus non-COVID-19 posts. Statistical analysis was performed with SPSS software (IBM SPSS Statistics for Mac, Armonk, USA). Frequencies between COVID-19 and non-COVID-19 posts were compared using chi-square tests with a p-value less than 0.05 considered significant.

## Results

A total of 563 posts were collected of which 354 were excluded based on the aforementioned criteria. The remaining 209 posts were all published by females at varying stages of IVF treatment including before (n=138, 66%), during (n=34, 16.3%), and after pregnancy (n=20, 9.6%), with pregnancy status unable to be determined in 8.1% (n=17). COVID-19 was mentioned in 106 posts (50.7%). Major themes and subthemes extracted from posts are detailed in Table 1. Statistical analysis demonstrated that the frequencies of posts between COVID-19 and non-COVID-19 posts significantly differed among the following themes and subthemes: barriers/setbacks, negative emotions, social support, and positive community offerings of social support (p < 0.05). 

**Table 1:** Frequency of Themes Described in March (n=209) *Denotes statistical significance between COVID-19 and non-COVID-19 posts defined by p<0.05.

Theme	Subtheme	Frequencies in COVID-19 posts (n=106)	Frequencies in non-COVID-19 posts (n=103)
Medical or physical experience		82.1% (87)	73.8% (76)
	Point in cycle	60.4% (64)	64.6% (67)
	Changes to body/side effects	5.7% (6)	13.5% (14)
	Barriers/setbacks*	51.9% (55)	10.7% (11)
Emotions		40.6% (43)	30.8% (32)
	Positive	6.6% (7)	12.5% (13)
	Negative*	24.5% (26)	12.5% (13)
	Conflicting	9.4% (10)	5.8% (6)
Social support*		67.0% (71)	39.8% (41)
	Positive family	6.6% (7)	12.5% (13)
	Positive religion	7.5% (8)	5.8% (6)
	Positive community support	17.0% (18)	13.5% (14)
	Positive community offering*	53.8% (57)	18.4% (19)
	Negative	4.7% (5)	3.8% (4)
Coping		35.8% (38)	30.8% (32)
	Self-care	8.5% (9)	7.7% (8)
	Positivity	17.9% (19)	11.7% (12)
	Religion	13.2% (14)	16.3% (17)
Education		5.7% (6)	13.5% (14)
	Requesting	3.8% (4)	7.7% (8)
	Offering	1.9% (2)	7.7% (8)

Posts describing the medical and physical experience of IVF were frequent (73.8-82.1%). Within these, three subthemes emerged including a description of point in the cycle (defined as posts documenting a time in pregnancy or treatment process), setbacks to care, and changes to the body (including side effects of treatment). COVID-19 was identified as a notable setback as many shared experiences following the ASRM’s cycle cancellation recommendation (Table 1). Posts describing setbacks expressed concerns with cycle cancellations, the delay in treatment, and new protocols that prohibited partners from attending appointments, procedures, and births.

The emotional spectrum pertaining to the IVF experience was another predominant theme and was divided into three subthemes: positive, negative, and conflicting emotions. Positive emotional expression including feelings of happiness, excitement, and gratefulness was exhibited more frequently by users whose treatment was unaffected by COVID-19. In contrast, negative emotions including feelings of sadness or disappointment often stemmed from patient concerns and worries about how the COVID-19 pandemic could affect their pregnancy. Instagram users were not only worried about how the virus could affect fetal health but also expressed feelings of being overwhelmed and fearful of the unknowns of the pandemic. Patients noted that despite the pause on cycles, their maternal clock had not stopped. This created an additional negative emotion of helplessness as they were dependent on infertility treatments to fulfill their dreams of building a family. Lastly, conflicting emotions were identified using posts with terms such as “nervous but excited” or “anxious but scared.” Conflicting emotions often stemmed from feelings of uncertainty relating to the COVID-19 pandemic.

Instagram users detailed various sources of social support including family, religion, and community members. Community support offered to others was commonly documented. Many users including those experiencing cycle cancellations and those who had previously undergone IVF treatment expressed sympathy and support to individuals facing treatment delays. Negative community support was also discussed with the main focus on a potential “baby boom” nine months following the quarantine period. Additionally, negative support was emphasized by one user who discussed her disappointment when she was told “welcome to the club” after her cycle had been canceled. Her post highlighted her eight-year struggle with infertility and the negative emotional impact experienced when others attempted to justify the cancellation. 

Women also authored posts about coping mechanisms utilized throughout the IVF process. The most common coping mechanisms were related to self-care, positivity, and religion. Coping posts referencing COVID-19 often utilized phrases such as “placing their trust in God” or “praying to God for success.” Posts are also discussed using self-care (e.g., exercise) to cope with the possibility of cycle cancellations or other common IVF stressors, such as the possibility of a failed cycle. Many women utilized coping through positivity (e.g., hoping for good news about their cycle) to overcome unforeseen circumstances and setbacks encountered due to the virus. 

Women used Instagram as a platform to offer knowledge or request advice about the IVF process. Educational posts specifically referencing COVID-19 were minimal. One post provided advice about what to do during uncertain times, while another post requested advice regarding choosing an obstetric practice; this post referenced analyzing practices based on the abundance of COVID-19 information provided on clinic websites. Further, one woman educated the IVF community about virus-specific policy changes in New York, namely the ban of visitors during deliveries, and requested the community sign a petition to the governor.

## Discussion

Understanding the patient experience during a global pandemic is imperative especially given the increased rates of mental health conditions (e.g., anxiety, depression) within the IVF community [5]. In our study, we selected Instagram posts that followed the ASRM’s recommendation to postpone IVF cycles to detail the experiences of patients impacted by COVID-19. Despite selecting posts after March 17, not all posts discussed COVID-19 as some cycles were allowed to continue if in the later stages of treatment and some women had already achieved pregnancy through IVF. The majority of patients impacted discussed setbacks and barriers to care through policy recommendations and clinic closures consistent with the ASRM’s cycle cancellation recommendation. In addition, posts authored by women impacted by COVID-19 contained more negative emotions and fewer positive emotions compared to those who were unaffected. Offerings of support to others significantly increased in posts mentioning COVID-19, highlighting the resilience and strength of the IVF community. Despite the observed increase in setbacks and negative emotions, posts offering support nearly tripled in frequency. To combat setbacks during the time of COVID-19, coping through positivity also increased. Given these findings, future studies should consider evaluating the potential use of formalized support groups on social media to improve care for IVF patients. These support groups could also be utilized by patients considering IVF treatments to gain insight into patient experiences, preview the community environment, and receive educational materials. The ability of online communities to mitigate the effects of pandemic-related changes and stress should also be explored in future studies. 

This study was limited by several factors including the inability to collect complete demographic information from participants on a single social media platform. Our study design was additionally limited to public posts, one hashtag and three dates, which some may argue does not fully capture the active landscape of social media. Lastly, in this patient-centered study, provider posts were excluded. Future exploration is warranted regarding how providers can interact with patients on social media to further support and educate the IVF community. Despite these limitations, this study introduced a novel approach to improve the understanding of IVF patient experiences. Future studies should similarly consider utilizing social media as a database to capture a candid account of patient beliefs, emotions, and events.

## Conclusions

Our thematic analysis supports the need for careful consideration of the psychological and social effects of cycle cancellations on the IVF community. Several posts highlighted the time-sensitive nature of IVF treatment. Posts detailed added stress for women of advanced maternal age and those who had spent years of time and large sums of money to prepare for the now-canceled cycle. Though there were many uncertainties surrounding the global pandemic caused by COVID-19, experiences and sentiments revealed by this study should be considered when a successive pandemic or global emergency threatens IVF treatment protocols.
